# Synergistic Effect of Oleanolic Acid on Aminoglycoside Antibiotics against *Acinetobacter baumannii*

**DOI:** 10.1371/journal.pone.0137751

**Published:** 2015-09-11

**Authors:** Bora Shin, Woojun Park

**Affiliations:** Laboratory of Molecular Environmental Microbiology, Department of Environmental Science and Ecological Engineering, Korea University, Seoul, Republic of Korea; Second University of Naples, ITALY

## Abstract

Difficulties involved in treating drug-resistant pathogens have created a need for new therapies. In this study, we investigated the possibility of using oleanolic acid (OA), a natural pentacyclic triterpenoid, as a natural adjuvant for antibiotics against *Acinetobacter baumannii*. High concentrations of OA can kill cells, partly because it generates reactive oxygen species. Measurement of the fractional inhibitory concentration (FIC) for OA and time-kill experiments demonstrated that it only synergizes with aminoglycoside antibiotics (e.g., gentamicin, kanamycin). Other classes of antibiotics (e.g., ampicillin, rifampicin, norfloxacin, chloramphenicol, and tetracycline) have no interactions with OA. Microarray and quantitative reverse transcription-PCR analysis indicated that genes involved in ATP synthesis and cell membrane permeability, the gene encoding glycosyltransferase, peptidoglycan-related genes, phage-related genes, and DNA repair genes were upregulated under OA. OA highly induces the expression of *adk*, which encodes an adenylate kinase, and *des*6, which encodes a linoleoyl-CoA desaturase, and deletion of these genes increased FICs; these observations indicate that *adk* and *des*6 are involved in the synergism of OA with aminoglycosides. Data obtained using 8-anilino-1-naphthalenesulfonic acid, fluorescence-conjugated gentamicin, and membrane fatty acid analysis indicates that *adk* and *des*6 are involved in changes in membrane permeability. Proton-motive force and ATP synthesis tests show that those genes are also involved in energy metabolism. Taken together, our data show that OA boosts aminoglycoside uptake by changing membrane permeability and energy metabolism in *A*. *baumannii*.

## Introduction

Public health risk caused by multidrug-resistant (MDR) bacteria has become a serious problem on a global scale. Efforts for treating MDR bacteria continue to be in the forefront of the clinician's practice in caring for victim of nosocomial infection. During the last decade, *Acinetobacter baumannii* has emerged as a major cause of healthcare-associated infections and nosocomial infections. *A*. *baumannii* is an important pathogen in healthcare settings, where it causes infections such as bacteremia, pneumonia, and meningitis, as well as urinary tract and wound infections [1–4]. The antibiotic resistance of *A*. *baumannii* isolates is an issue of particular concern, and the molecular mechanisms underlying this resistance are poorly understood. First-line treatment of *A*. *baumannii* infections requires the administration of a carbapenem antibiotic, but resistance is increasingly common. Other therapeutic options include aminoglycosides, sulbactam, polymyxins, and tigecycline [5]. Of these, aminoglycosides comprise a class of antibiotics that can be used to treat carbapenem-resistant *A*. *baumannii* infections. In bacteria, aminoglycosides bind to ribosomal RNA and induce mistranslation, which leads to the inhibition of protein synthesis and thus cause bacterial death [6]. Aminoglycosides are highly potent, broad-spectrum antibiotics that have been traditionally used for the treatment of serious gram-negative infections [6, 7]. They kill bacteria by inhibiting protein synthesis via binding to 16S rRNA and by disrupting bacterial cell membrane integrity [8]. Aminoglycosides combined with β-lactam antibiotics, which inhibit cell wall synthesis, may be the most reliable therapy for the treatment of MDR *Enterococcus* species [9]. However, side effects such as ototoxicity, nephrotoxicity, and organ failure remain serious problems resulting from the clinical use of aminoglycosides [10]. In addition, many isolates of pathogenic *A*. *baumannii* are resistant to one or more members of this class [6–7]. Thus, it is necessary to find novel therapies with fewer side effects and greater therapeutic action than the options currently available.

Oleanolic acid (OA) and ursolic acid (UA) are triterpenoid compounds that exist widely in food, medicinal herbs, and other many plants. Members of the triterpenoid family include betulinic acid and triterpenoid saponins. The therapeutic activities of OA and other triterpenoids have been described in numerous reports [11–14]. OA can be used for the treatment of human liver disorders and for the synthesis of triterpenoid saponins [11]. Glossy privet fruit and hawthorn fruit are two herbs commonly used in Chinese medicine [15] and have antioxidative effects which have been ascribed to triterpenoid components such as OA and UA [16, 17]. It has been reported that triterpenoid family compounds including OA and UA have hepatoprotective, antitumor, and weak anti-HIV and anti-HCV activities [12]. In addition, OA modulates enzymes that act in insulin biosynthesis, secretion, and signaling [18]. Recent study described that OA has been shown to reduce obesity by improving glucose tolerance in mice [19]. Moreover, in eukaryotic cells, OA is involved in activating the transcription factor nuclear factor erythroid-2-related factor-2 (Nrf2) [18, 20]. Triterpenoids also are potent inhibitors of various pathogenic bacteria [21–23] and act as protective compounds in eukaryotic cells [14, 24], but there is a lack of information on the mechanisms underlying this antibacterial activity.

Many studies have shown that the efficiency of antibiotics is sometimes improved by the addition of other compounds that are involved in synergism. [25–27]. According to a recent study, chimeric peptides have synergistic effects with antibiotics in reducing biofilm formation and bacterial growth [28]. In this study, we investigated the synergistic effects of OA when administered in combination with commercial antibiotics. Transcriptome analysis showed that OA is involved in membrane permeability, energy synthesis, and ribosomal protein synthesis. Our findings indicate that OA acts synergistically in combination with aminoglycosides to increase energy, membrane permeability, and ribosomal protein production.

## Materials and Methods

### Bacterial strains and chemicals

The bacterial strains used in these experiments are *A*. *baumannii* ATCC17978 (GenBank accession no. CP000521) and its mutants, Δ*adk* and Δ*des6*, which were constructed for this study. All the strains were grown for 14 h in Luria-Bertani (LB) broth at 37°C with rotational shaking at 220 rpm. Tetracycline, chloramphenicol, norfloxacin, rifampicin, carbonyl cyanide-m-chlorophenyl hydrazone (CCCP), dihydrorhodamine 123 (DHR123), and 8-anilino-1-naphthalenesulfonic acid (ANS) were purchased from Sigma-Aldrich. Kanamycin and ampicillin were purchased from Fluka and Amresco. Gentamicin sulfate and Texas red (TR) were purchased from Invitrogen. OA was purchased from Wako (Japan). Each antibiotic was diluted to the appropriate concentration. Minimum inhibitory concentration (MIC) was used and each subsequent concentration was reduced by half. OA was dissolved in ethanol to 5 mg/ml and stored at 4°C.

### Measurement of fractional inhibitory concentrations

The antibacterial activity of antibiotics in combination with OA was assessed by the microdilution checkerboard method [25]. Overnight cultures of *A*. *baumannii* ATCC17978 were diluted with fresh LB media to a final concentration of 10^6^ CFUs/ml, and 200 μl aliquots were placed on each well in 96-well plates. The concentrations of antibiotics used in microdilution checkerboard method were 0.06 to 16 μg/ml for kanamycin, 0.01 to 2 μg/ml for gentamicin, 2 to 512 μg/ml for ampicillin, 0.005 to 0.5 μg/ml for tetracycline, 0.25 to 64 μg/ml for chloramphenicol, 0.03 to 8 μg/ml for norfloxacin and 0.02 to 4 μg/ml for rifampicin. After adding the antibiotics to each plate, OA (4–256 μg/ml) was added to each well, with the same volume of ethanol used as the negative control. Plates were incubated at 37°C for 24 h and read using a microplate spectrophotometer (Bio-Tek). The fractional inhibitory concentration (FIC) of an agent was determined as the MIC of the combination of OA and an antibiotic divided by the MIC of the agent (e.g., OA or the antibiotic) alone. The FIC index (FICI) was calculated as the sum of the FICs of OA and the antibiotic in question; an FICI ≤0.5 indicates synergy, an FICI >0.5 but ≤4 indicates no interaction, and an FICI >4 indicates antagonism [26].

### Oxidative stress detection using flow cytometry

Measurement of reactive oxygen species (ROS) was performed by using DHR123 as previously described [29]. *A*. *baumannii* ATCC17978 cells in the exponential phase of growth were treated for 1 h with 32 μg/ml or 64 μg/ml OA dissolved in ethanol. Then, 2 ml of the treated cells was washed in phosphate-buffered saline (PBS). Next, 5 μg/ml DHR123 was added to the cells, which were subsequently incubated at 37°C for 1 h in the dark. The cells were then washed three times with PBS and then resuspended in 1 ml of PBS and transferred to flow cytometry tubes. ROS levels were measured using a FACSVerse Flow Cytometer (BD Biosciences) and quantified by determining the mean of the fluorescence intensity of each treatment. Three independent experiments were conducted for each condition, with typically 10,000 cells analyzed per experiment, using BD Cell Quest Pro software (BD Biosciences).

### Microarray analysis

*A*. *baumannii* ATCC17978 cells in the exponential phase of growth were treated for 30 min at 37°C with 64 μg/ml OA dissolved in ethanol. Microarray analysis was performed as previously described [30]. RNA was isolated using the RNeasy Mini kit (Qiagen), and RNA concentrations were estimated by measuring absorbance at 260 nm. The cDNA probes for microarray analysis were prepared by reverse transcription of total RNA (50 μg). The cDNA probes were cleaned up using a Microcon YM-30 column (Millipore) and then coupled to Cy3 or Cy5 (GE Healthcare). The dried Cy3- or Cy5-labeled cDNA probes were then resuspended in 55μl of 2x HI-RPM hybridization buffer (Agilent) containing 30% formamide (v/v), 5× saline-sodium citrate, 0.1% SDS (w/v), and 0.1 mg/ml salmon sperm DNA. The Cy3- or Cy5-labeled cDNA probes were mixed together and hybridized onto a microarray chip (MYcroarray.com). The hybridization images on the slides were scanned using an Axon 4000B (Axon Instruments), and data quantification was performed using GenePix Pro 6.0 (Axon Instrument). Genes that were upregulated more than 1.5-fold in at least two replicates were selected. The microarray data were deposited in the National Center for Biotechnology Information GEO site (under accession number GSE 60239). Microarray data were confirmed by quantitative reverse transcription PCR (RT-PCR).

### Quantitative RT-PCR analysis

To confirm the microarray results, the following eight genes were selected at random from the set of genes that had been found to be highly expressed in the microarray analysis for further investigation by quantitative RT-PCR: A1S_0092, A1S_2016, A1S_3398, A1S_0152, A1S_0041, A1S_0080, A1S_1023, and A1S_0058. The cDNA was produced using an aliquot of the same RNA that had been used for the microarray analysis. The PCR mixture contained 12.5 μl of iQ SYBR Green Supermix (Bio-Rad), 1 μl of each primer (0.5 mM), and 1.5 μl (1 mg) of cDNA, comprising a total volume of 25 μl. The PCR was conducted under initial denaturation conditions of 95°C for 3 min followed by 40 cycles of 45 s at 95°C, 45 s at 60°C, and 45 s at 72°C. The quantification results were derived from triplicate samples.

### Construction of *A*. *baumannii* ATCC17978 Δ*adk* and Δ*des6* mutants

The construction of the Δ*adk* and Δ*des6* mutants was performed using a previously described procedure [30]. The genes *adk* (A1S_1023), which encodes adenylate kinase, and *des6* (A1S_0041), which encodes a putative linoleoyl-CoA desaturase, were amplified using the following primers: *adk*_KO_F (5ʹ-CGCGAATTCAGTTTCAGATGAACTCATTATCGGT-3ʹ) and *adk*_KO_R (5ʹ-CGCGGTACCTTTCACCAGAAGCAGCACGA-3ʹ) for *adk*, and *des6*_KO_F (5ʹ-CGCGAATTCGATGCGGCTAAGGTCCGTAA-3ʹ) and *des6*_KO_R (5ʹ-CGCGGTACCTTTATAGCGGTTGGCAGGCA-3ʹ) for *des6*. The resulting PCR products were digested with *Eco*RI and *Kpn*I restriction enzymes. Each fragment was subsequently cloned into a pVIK112 vector. The constructed plasmids were then transformed into *Escherichia coli* strain S17-1λ pir. For each plasmid, the transformed cells were transferred to an LB agar plate containing kanamycin, and a single colony was selected for subsequent growth and plasmid extraction. The resulting plasmid was transformed into *A*. *baumannii* ATCC17978 via electroporation. Conjugation was performed using the biparental filter mating method with the ATCC17978 strain. The resulting transformants (Δ*adk* and Δ*des6*) were selected in LB agar medium containing 50 μg/ml kanamycin.

### Growth measurement

Overnight cultures of ATCC17978, Δ*adk*, and Δ*des6* were grown at 37°C in LB medium. One milliliter of cells was collected and washed twice with PBS, and 10^6^ CFUs/ml were inoculated in 50 ml LB and grown for 13 h with vigorous aeration (220 rpm). Growth was monitored by measuring the OD_600_ of the cultures using a BioPhotometer (Eppendorf).

### ANS fluorescence uptake

To assess the integrity of bacterial cell membranes under OA treatment, an ANS probe was used as follows [31]. ANS stock solutions (5 mg/ml) were prepared in dimethylformamide and stored in the dark at 4°C. The cells were grown to the exponential phase at 37°C in 5 ml LB media with aeration. Then, the cells were treated with OA for 1 h. To measure fluorescence, 10 μM ANS was added to the multiwell plates containing cells that had been washed twice in PBS. The spectrum of ANS in the absence of cells was used as a control. ANS fluorescence emission spectra were recorded at wavelengths ranging from 385–400 nm with excitation at 350–380 nm using the VICTOR3 microtiter plate reader (Bio-Rad).

### Microscopic analysis and quantification of fluorescence

Conjugation of gentamicin and Texas-red (TR) was performed as described in a previous study [32]. Briefly, 4.4 ml of 50 mg/ml gentamicin was mixed with 0.6 ml of 2 mg/ml TR esters to produce a 300:1 molar ratio of gentamicin:TR and incubated for 12 h at 4°C. The resulting conjugates had molecular weights of 1165, 1179, and 1193 g/mol after the loss of the carbonyl amine from the reactive TR. Gentamicin has three or four amine groups depending on its isoform, and the conjugation of a TR molecule to a gentamicin amine group reduces the ionic charge of the conjugated molecule by 1 for each amine group generally one) conjugated to TR, proportionately increasing its hydrophobicity. We then confirmed the effect of the gentamicin-TR conjugate (GTTR) on ATCC17978, Δ*adk*, and Δ*des*6. Each strain was grown to the exponential phase, and then the cells were harvested by centrifugation at 13,200 rpm for 1 min and washed twice in PBS. The cells were then resuspended in 1 ml PBS and transferred to a 1.5 ml microfuge tube. Then, the cells were incubated with 300 μg/ml GTTR for 30 min at 37°C, after which they were washed twice in PBS. The resuspended cells were then placed in polystyrene 48-well microtiter plates (Costar), and the GTTR fluorescence intensity was quantified using a microtiter plate reader (Hidex) with an excitation wavelength of 535 nm and an emission wavelength of 618 nm. The effect of different growth rates on fluorescence intensity was excluded by normalizing the absorbance to the OD_600_ value. One fluorescence unit was defined as the difference between the fluorescence intensity of the cells and the fluorescence intensity of PBS divided by the OD_600_ of the cells. For the fluorescence microscopy experiment, cells were viewed with an Axio Lab microscope (Zeiss).

### Fatty acid methyl esters analysis

Cellular fatty acids were extracted from ATCC17978, Δ*adk*, and Δ*des6*, transformed into fatty acid methyl esters (FAMEs) [33], and then analyzed via gas chromatography using MIDI calibration standards, as follows. FAMEs were prepared by saponification and methylation of fatty acids cleaved from the lipids. The extracted FAMEs were then analyzed using an Agilent 7890 GC system with a flow ionization detector and an HP-Ultra-2 capillary column (crosslinked 2.5% phenylmethyl silicone, 25 m, 200 mm i.d., film thickness: 0.33 mm). The FAMEs were identified and qualified using the Sherlock 6.0B MIDI software according to their equivalent chain value.

### Determination of ATP concentration

To measure intracellular ATP concentrations, the ENLITEN ATP Assay System Bioluminescence Detection Kit (Promega) was used in accordance with the manufacturer’s instructions. Cultures of ATCC17978, Δ*adk*, and Δ*des6* were grown to the exponential phase and then treated with 64 μg/ml OA for 30 min. Next, the cells were harvested and then resuspended in 1% trichloroacetic acid (TCA) buffer. Prior to the assay, the samples were neutralized by diluting them fivefold with 250 mM Tris-acetate buffer (pH 7.75). The luminescence was measured using a microplate reader (Hidex).

### Measurement of membrane potential

We used the BacLight Bacterial Membrane Potential Kit (Invitrogen) to assess changes in proton-motive force (PMF) according to the kit protocol, as follows. Briefly, cells were harvested from 2 ml of cultures that had been grown to the exponential phase; the cells were then resuspended in 1 ml of PBS without washing. Then, 10 μM DiOC_2_(3) was added to the cells, which were then incubated for 30 min at room temperature. Immediately before the analysis, 1 ml each of cells and PBS were added to the flow tubes. The samples were analyzed on a FACSVerse Flow Cytometer (BD Biosciences) with settings optimized according to the kit manual. The settings used were FITC (green) and PerCP-Cy5.5 (red), and 10,000 events were recorded. For each strain, the red/green (PerCP-Cy5.5/FITC) value was determined, compared to the depolarized control sample treated with CCCP which destroys membrane potential by eliminating the proton gradient.

### Time-kill assay

The 2-fold MIC of gentamicin and CCCP were used to determine the survival rates of ATCC17978, Δ*adk*, and Δ*des6* under stress conditions. For each strain, cells were harvested from 5 ml of culture grown to the exponential phase. The cells were washed three times in autoclaved PBS, and then the cells were inoculated at 10^7^ CFUs/ml into a 100 ml flask containing 30 ml of PBS supplemented with either gentamicin (4 μg/ml) or CCCP (10 μM). The cultures were then incubated at room temperature with constant agitation (220 rpm). At each time point (0, 5, 10, 15, and 30 min), cells were harvested and washed twice in PBS; then, 20 μL aliquots were transferred onto LB agar plates for colony counting. Cell survival was determined from the relative percentage of CFUs/ml according to the following equation: (CFU at the indicated times/CFU for samples incubated for 0 h after adding antibiotics) × 100.

### NBT assay

Superoxide anion production during treatment of OA and solvent was determined using NBT (nitro blue tetrazolium) as described previously [34]. One milliliter of cells in exponential phase was incubated with OA and ethanol for 30min and 0.5 mL of 1mg/ml NBT was added for 30 min at 37 C°. Then, 0.1 mL of 0.1 M HCl was added, and the tubes were centrifuged at 1,500xg for 10 min. The separated pellets were treated with 0.4 mL of dimethyl sulfoxide to extract the reduced NBT; finally, 0.8 mL of PBS (pH 7.5) was added, and the absorbance was measured at 575 nm. OD_575_ of superoxide production was measured by spectrophotometer. Each experimental point represents the mean of three replicates.

## Results

### Evaluation of the synergistic effects of combinations of OA and conventional antibiotics on *A*. *baumannii*

To evaluate possible synergisms between OA and antibiotics, the checkerboard method was performed using *A*. *baumannii* ATCC17978. We found that OA reduced the MICs of aminoglycosides (e.g., gentamicin, kanamycin) but did not affect the MICs of other types of antimicrobial agents (Table 1). The MIC of OA alone against *A*. *baumannii* ATCC17978 was greater than 512 μg/ml, and thus its antibacterial activity was very low. However, the MICs of gentamicin and kanamycin were remarkably reduced in the presence of OA (Table 1). The MIC of kanamycin and gentamicin in combination with OA decreased to 1/4 of the MIC of alone; from 16 μg/ml to 4 μg/ml and from 2 μg/ml to 0.5 μg/ml, respectively. To evaluate the synergistic effects of OA, FICI values, calculated as the sum of the FICs for OA and the antibiotic in question. Using this protocol, we found that the combination of OA with gentamicin and kanamycin gave FICI values of 0.375 and 0.313, respectively, against ATCC17978; these values are indicative of synergism. When tetracycline was used, FICI value appears to be 0.531 and FICIs of other antibiotics ranges from 0.625 to 1.000. Measurement of FIC index has limitations and careful interpretation is necessary [35]. Time-kill assay was conducted to measure actual synergistic activity of OA with aminoglycosides (S1 Fig). Bactericidal effect was much higher under combination of gentamicin 1/16 MIC (0.13 μg/ml) and OA <1/16 MIC (64 μg/ml) than gentamicin 1/8 MIC (0.25 μg/ml) alone (S1 Fig).

**Table 1:** Interactions between antibiotics and oleanolic acid as determined by the fractional inhibitory concentration index in *Acinetobacter baumannii* ATCC17978.

Agents	MIC (μg/ml)		FIC^b^	FICI^c^	Outcome
	Alone	Combination^a^			
**Kanamycin**	16.00	4.00(64)	0.250(0.125)	<0.375	Synergy
**Gentamicin**	2.00	0.50(32)	0.250(0.063)	<0.313	Synergy
**Ampicillin**	512.00	128.00(256)	0.250(0.500)	<0.750	No interaction
**Tetracycline**	0.50	0.25(16)	0.500(0.031)	<0.531	No interaction
**Chloramphenicol**	64.00	8.00(256)	0.125(0.500)	<0.625	No interaction
**Norfloxacin**	8.00	1.00(256)	0.125(0.500)	<0.625	No interaction
**Rifampicin**	4.00	2.00(256)	0.500(0.500)	< 1.000	No interaction

MIC, minimum inhibitory concentration. The MIC concentration of oleanolic acid was >512 μg/ml under alone treatment.

^a^The number in parentheses indicates the MIC of oleanolic acid of the combination.

^b^FIC, fractional inhibitory concentration; FIC was calculated as MIC of the combination divided by the MIC of the agent alone. The FIC index of oleanolic acid was shown in parentheses.

^c^FICI, FIC index; calculated as the sum of the FICs of oleanolic acid and gentamicin.

### Induction of oxidative stress by OA

**Fig 1:**
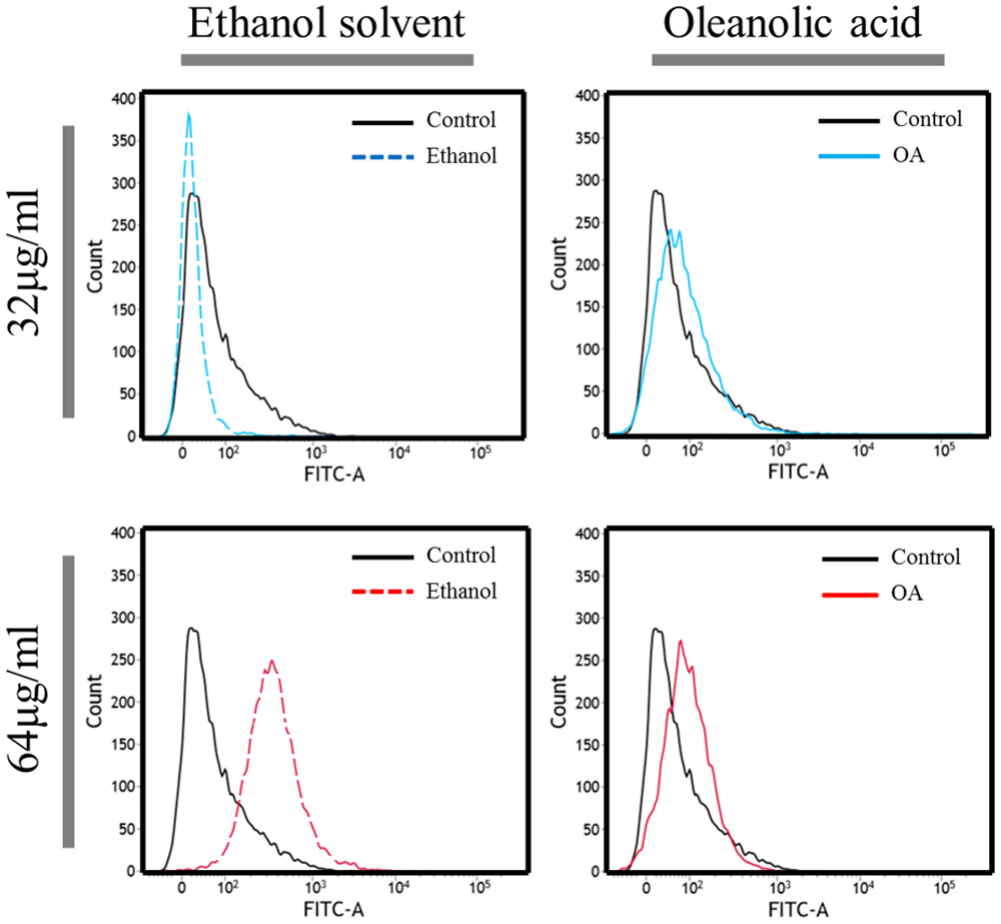
Generation of reactive oxygen species by oleanolic acid in *Acinetobacter baumannii* ATCC17978. Flow cytometry analysis was conducted using dihydrorhodamine dye (5 mg/ml) to measure oxidative stress in response to oleanolic acid (OA). After exposure for 1 h to OA, the cells were washed twice in phosphate-buffered saline and placed in Falcon FACS tubes for flow cytometry analysis. Flow cytometry analysis of cells treated with 32 μg/ml of OA (indicated with a solid blue line) and cells treated with an equivalent volume of ethanol (indicated with a red dashed line). And flow cytometry analysis of cells treated with 64 μg/ml of OA (indicated by the solid green line) and cells treated with an equivalent volume of ethanol (indicated with a blue dashed line).

We conducted a flow cytometry assay using the ROS dye DHR123, which was used as an indicator of intracellular oxidative stress. This uncharged, nonfluorescent compound can diffuse passively across most uncharged membranes, and oxidation converts it to a fluorescent product [29]. Superoxide anion produced during growth with OA was determined using NBT (nitro blue tetrazolium) assay [34]. Because the OA is dissolved in ethanol, we compared oxidative stress levels in cells treated with OA to cells treated with ethanol alone, at various concentrations ranging from 8 μg/ml to 128 μg/ml. But, at the lowest concentration of OA (8 μg/ml), we did not observe a difference between the level of oxidative stress in cells that were treated with OA and that in cells treated with ethanol (data not shown). On the other hand, we found that there was a difference in the level of ROS formation between cells treated with OA and cells treated with ethanol when the OA concentration was increased to 32 μg/ml and 64 μg/ml (Fig 1). Growth inhibition was observed with 64 μg/ml OA in *A*. *baumannii*, but the same volume of ethanol displayed a little growth defect (S2 Fig). When higher concentration of OA (128 μg/ml) was used, ethanol solvent alone could inhibit the growth, thus further experiments were conducted using OA concentration below 128 μg/ml. Consistent with our flow cytometry assay, more superoxide production was measured using the NBT assay under high concentration of OA (S3 Fig).

### Transcriptome analysis of *A*. *baumannii* under OA treatment

To find the mechanism underlying the synergistic effect of OA, microarray analysis was performed on *A*. *baumannii* ATCC17978 cells treated with OA. Our analysis demonstrated that 250 genes were differentially expressed by more than 1.5-fold (227 genes upregulated and 23 genes downregulated (accession number GSE 60239, data not shown). Interestingly, OA induced many genes involved in the oxidative stress response, the cell membrane, transport and transferase, energy synthesis, cell shape, and the ribosome (Table 2).

**Table 2:** *Acinetobacter baumannii* ATCC17978 genes indicated by microarray analysis as being upregulated after exposure to oleanolic acid.

Locus tag	Gene	Protein name	Fold change
**Oxidative stress, DNA repair**			
A1S_0617	*ohr*	Predicted redox protein, regulator of disulfide bond formation	1.658
A1S_2717		Dihydrolipoamide dehydrogenase	1.657
A1S_0315		DNA repair system	1.629
A1S_0159	*gpx*	Glutathione peroxidase	1.597
A1S_2664	*groEL*	60 kDa Chaperonin	1.518
**Cell envelope biogenesis, outer membrane**			
A1S_0041	*des6* ^a^	Putative linoleoyl-CoA desaturase	1.673
A1S_0058	*gtfs* ^a^	Glycosyltransferase	1.891
A1S_3398	*murl* ^a^	Glutamate racemase	1.675
A1S_0311	*ybaW* ^a^	Putative acyl-CoA thioesterase II	1.581
A1S_0431	*htrB* ^a^	Lipid A biosynthesis lauroyl acyltransferase	1.503
A1S_2078	*yhbS* ^a^	Predicted acetyltransferase	1.504
A1S_3406	*hutU* ^a^	Urocanate hydratase	1.807
A1S_0494	*gtfs*	Putative glycosyl transferase	1.623
A1S_2016	*nucD*	Lysozyme, Phage-related lysozyme	1.701
A1S_2089	*yehB* ^a^	Fimbrial usher protein	1.561
A1S_0232		Type 4 fimbriae expression regulatory protein	1.509
A1S_0329	*pilB* ^a^	Type 4 fimbrial biogenesis protein	1.552
**Energy metabolism**			
A1S_1023	*adk*	Adenylate kinase	2.416
A1S_0152	*atpH*	Membrane-bound ATP synthase F1 sector,	1.675
A1S_0080	*fabB* ^*a*^	Beta-ketoacyl-ACP synthase I	1.663
A1S_0155	*atpD*	ATP synthase subunit beta	1.626
A1S_0151	*atpE*	Membrane-bound ATP synthase F0 sector	1.539
A1S_0153	*atpA*	ATP synthase subunit alpha	1.513
A1S_1443	*tauB*	Taurine ATP-binding transport system component	1.506
A1S_0587	*panC*	Pantothenate synthetase	1.523
**Transport, transferase**			
A1S_0092	*foxA* ^a^	Putative ferric siderophore receptor protein	1.743
A1S_0538	*macA* ^a^	Putative RND drug transporter	1.540
A1S_0651	*traB*	TraB protein	1.626
A1S_0029	*ssuA* ^a^	ABC-type nitrate/sulfonate/bicarbonate transport system	1.514
A1S_0922	*hmt-1* ^a^	Putative homocysteine S-methyltransferase protein	1.667
A1S_3198	*rlmA1* ^a^	23S Ribosomal RNA G745 methyltransferase	1.542
A1S_3244	*thrB*	Homoserine kinase	1.532

^a^ Gene names derived from the KEGG orthology; the similarity to these genes is more than 70%

Among oxidative stress genes, genes encoding a predicted redox protein (A1S_0617; *ohr*), dihydrolipoamide dehydrogenase (A1S_2717), a DNA repair system protein (2OG-Fe(II)-oxygenase-like; A1S_0315), chaperonin (*groEL*), and glutathione peroxidase (*gpx*) were upregulated (Table 2). The redox protein encoded by A1S_0617 is predicted to be the organic hydroperoxide resistance protein (Ohr), which is a thiol-dependent peroxidase and is known to be important in the bacterial oxidative stress response [36]. Interestingly, Ohr is reduced by lipoyl-dependent systems [37]. Dihydrolipoamide dehydrogenase, encoded by A1S_2717, functions as an E3 component of a 2-oxoglutarate dehydrogenase multi-enzyme complex that might be responsible for reducing Ohr in *A*. *baumannii* ATCC17978 cells.

OA-induced proteins involved in general membrane and cell envelope biogenesis could be linked to membrane permeability. OA induced the expression of the gene encoding linoleoyl-CoA desaturase, which appears to produce unsaturated fatty acids for membranes. Glycosyltransferase, encoded by A1S_0058, may control the biosynthesis of glycans and glycoconjugates and thus might play an essential role in exopolysaccharide production [38]. Urocanate hydratase, encoded by A1S_3406, and lysozyme, encoded by A1S_2016, could be linked to the histidine degradation pathway [39] and cell wall degradation [40]. The expression of *adk* (A1S_1023) increased by 2.416-fold upon OA exposure. Adenylate kinase, encoded by *adk*, is involved in ATP turnover by catalyzing the reversible synthesis of ATP from ADP and ADP from AMP [41]. Several other ATP synthesis-related genes were also upregulated in response to OA treatment, and it may be that ATP synthesis increases under these conditions due to its role in cellular protection. It has been reported that the production of F_0_F_1_-ATP synthase was increased in a *Lactobacillus plantarum* strain under ethanol stress [42] and in a *Weissella confusa* strain under bile salt stress [43]. We found that 10 genes encoding 30S ribosomal proteins and 14 genes encoding 50S ribosomal proteins were upregulated (>1.5 fold change). Expression of a gene encoding the elongation factor EF-Ts (A1S_2322) increased by 1.592-fold (data not shown). The reason for the upregulation of the ribosomal and protein-elongation factor genes remains unclear.

**Fig 2:**
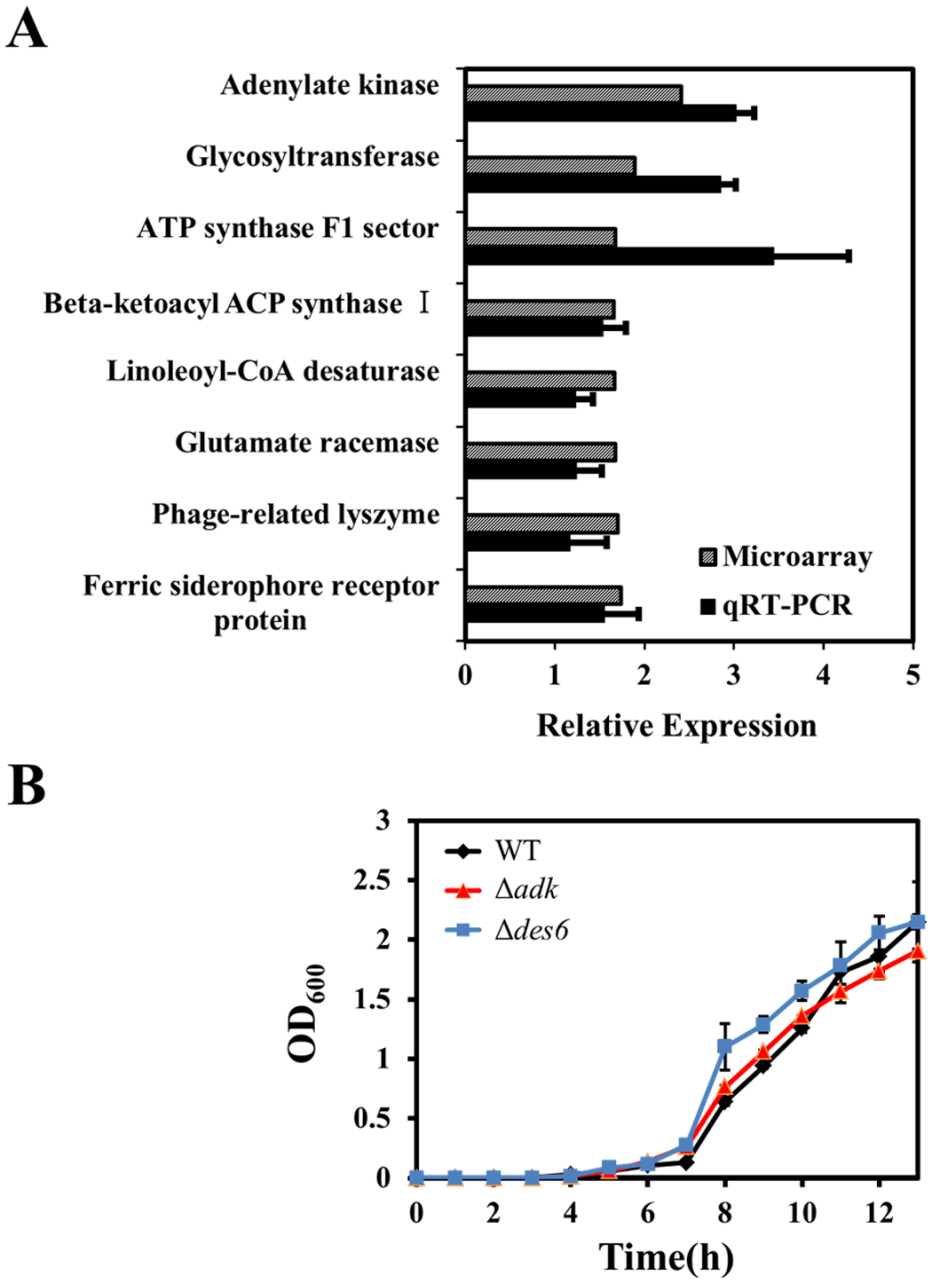
Genes upregulated in *Acinetobacter baumannii* ATCC17978 after treatment with oleanolic acid, and growth curves for mutants constructed on the basis of the microarray data. (A) RNA was extracted from *Acinetobacter baumannii* ATCC17978 cells grown to the exponential phase in Luria Broth after treatment with 64 μg/ml oleanolic acid for 30 min. The relative expression levels of eight genes as assessed by quantitative reverse transcriptase PCR (qRT-PCR; black bars) were consistent with the corresponding microarray data (gray bars). (B) Growth curves of wild-type *Acinetobacter baumannii* ATCC17978 and mutant strains for 13 h. Based on the results of the microarray data, we constructed Δ*adk* (red triangles) and Δ*des6* (blue squares) mutants; WT, wild-type strain; Δ*adk*, *adk* deletion strain; Δ*des6*, *des6* deletion strain. Data is represented as the average of three replicates.

From the genes that were highly expressed in the microarray, we randomly selected eight to confirm our microarray analysis by quantitative RT-PCR (Fig 2A): A1S_1023, A1S_0058, A1S_0092, A1S_2016, A1S_3398, A1S_0152, A1S_0041, and A1S_0080. Our microarray analysis was confirmed by the quantitative RT-PCR data. Two interesting genes, *adk* and *des6*, which are involved in ATP synthesis and membrane integrity, respectively, were chosen for further analysis, and their mutants were constructed. Interestingly, the synergistic effect of OA with aminoglycoside disappeared in Δ*adk* and Δ*des6* (Table 3), although the growth rates of these mutants were similar to that of the wild-type strain. (Fig 2B). These observations suggest that ATP synthesis and membrane integrity may be important factors that enable synergism between OA and aminoglycosides. Our complementation experiment for those deleted genes was unsuccessful, probably because of a plasmid effect or a requirement for fine-tuning of gene dosages [44, 45].

**Table 3:** Fractional inhibitory concentrations of oleanolic acid and gentamicin in wild-type *Acinetobacter baumannii* ATCC17978 and the mutant strains Δ*adk*, and Δ*des6*.

Strain	MIC (μg/ml)				FICI^a^	Outcome
	Alone		Combination			
	Oleanolic acid	Gentamicin	Oleanolic acid	Gentamicin		
**Wild-type**	512.00	2.00	32.00	0.50	0.313	Synergy
**Δ*adk***	512.00	0.50	32.00	0.25	0.563	No interaction
**Δ*des6***	512.00	2.00	256.00	0.50	0.750	No interaction

MIC, minimum inhibitory concentration. FIC, fractional inhibitory concentration; FIC was calculated as MIC of the combination divided by the MIC of the agent alone.

^b^FICI, FIC index; calculated as the sum of the FICs of oleanolic acid and gentamicin.

### The influence of OA on membrane permeability in *A*. *baumannii*

**Fig 3:**
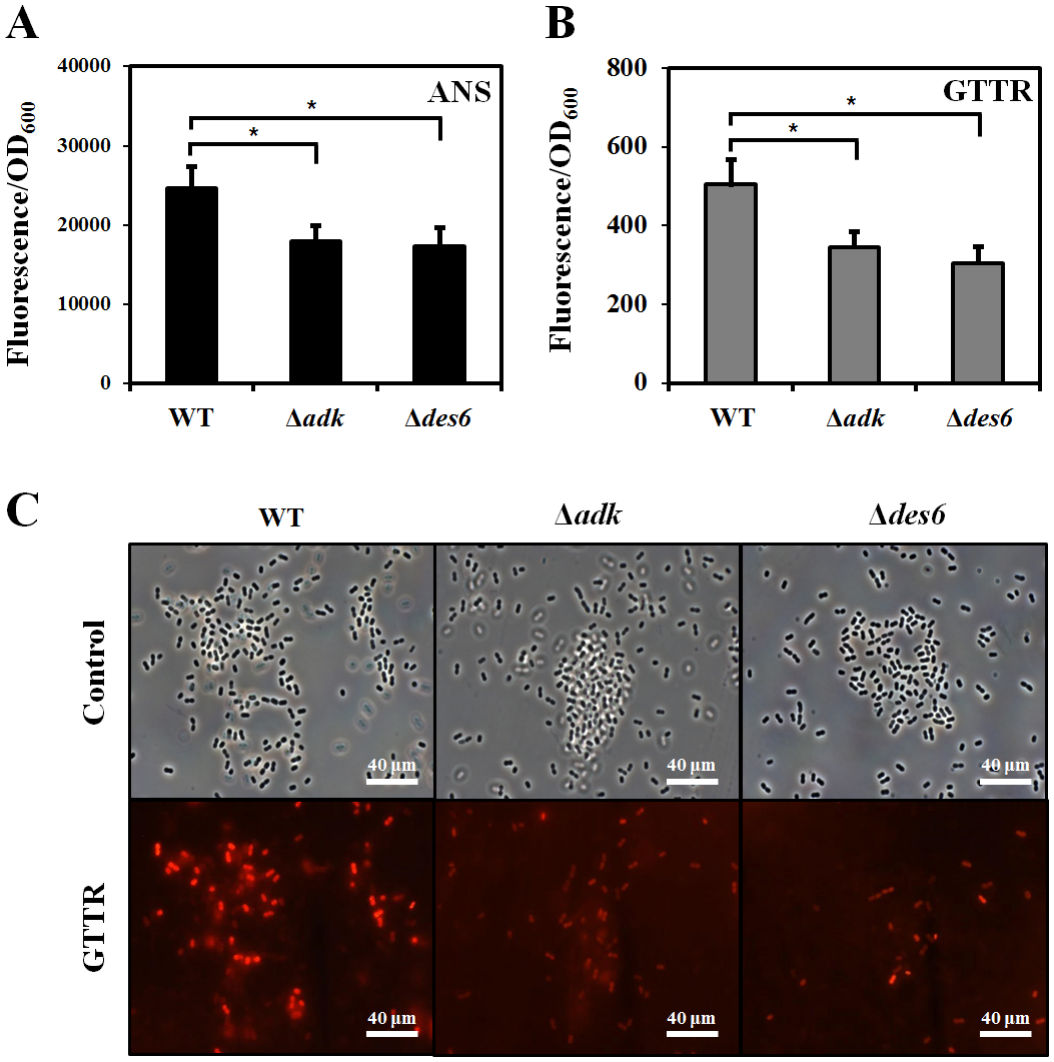
Measurements of membrane permeability by fluorescence uptake. (A) 8-Anilino-1-naphthalenesulfonic acid (ANS) staining in wild-type *Acinetobacter baumannii* ATCC17978 and the mutant strains Δ*adk* and Δ*des6*. After the cells were exposed to oleanolic acid (OA) for 1 h, 10 μM ANS was added, and absorbance was measured at 385–400 nm using excitation at 350–380 nm. (B) Gentamicin-Texas red (GTTR) uptake experiment. Cells were exposed to GTTR for 30 min and then washed in phosphate-buffered saline twice (C) Microscopy data confirm that wild-type cells uptake more GTTR than Δ*adk* or Δ*des6* cells do. All fluorescence values were normalized to OD_600_ values to compensate for differing rates of cell growth between wells. Error bars represent standard deviations of two replicates from the sample bacterial suspension and significance was measured by Student’s *t*-test (* P<0.05).

To measure the membrane permeability of cells, we used ANS, which is a neutrally charged, hydrophobic probe that fluoresces weakly in aqueous environments but exhibits enhanced fluorescence in nonpolar/hydrophobic environments, and which has been widely used to monitor changes in membrane permeability and protein conformation. We found that ANS fluorescence intensity was reduced in both Δ*adk* and Δ*des6* mutants as compared to that in the wild-type strain (Fig 3A); this observation indicates that the upregulation of these two genes in response to OA treatment might be important for making cells more permeable to antibiotics. We could not use OA with ANS together in our experiment because both molecules are hydrophobic and if used in combination would lead to cell aggregation [46]. Instead, fluorescently labeled gentamicin (GTTR) was used to investigate the uptake of aminoglycosides by cells. Cells were incubated with 300 μg/ml GTTR for 30 min and then washed twice, as described in a previous study [32]. More GTTR were taken up by the wild-type cells than by the mutants (*t*-test, p<0.05), although there was no significant difference in the amount of GTTR uptake between the Δ*adk* and Δ*des6* mutants (Fig 3B). These results were visualized by fluorescence microscopy (Fig 3C).

**Fig 4:**
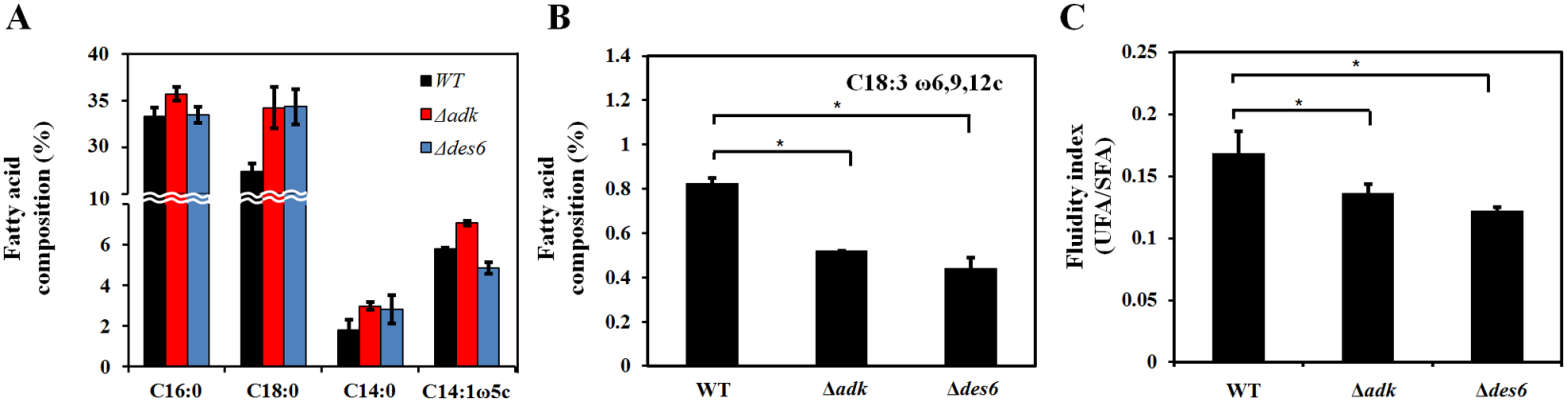
Effect of *adk* and *des6* on composition change and membrane fluidity. (A) Four major cellular fatty acids were calculated by percentage of each fatty acid in total fatty acids of wild-type (black), Δ*adk* cells (red), and Δ*des6* cells (blue). (B) Percentage of C18:3 ω6, 9, 12c (gamma linolenic acid) fatty acid. (C) Fluidity index calculated as total unsaturated fatty acid (UFA) divided by total saturated fatty acid (SFA). Error bars represent standard deviations of two replicates from the sample bacterial suspension and significance was measured by Student’s *t*-test (* P<0.05).

To measure the change in membrane composition in response to OA treatment, FAME analysis was performed. The main fatty acid components of all three strains were C16:0 (palmitic acid), C18:0 (stearic acid), C14:0 (myristic acid), and C14:1 ω5c (myristoleic acid). The amounts of C16:0, C18:0, C14:0, and C14:1 ω5c detected in wild-type cells were 32.6%, 20.9%, 1.8%, and 5.8%, in the Δ*adk* mutant cells were 37.5%, 34.5%, 3.0%, and 7.1%, and in the Δ*des6* mutant cells were 33.0%, 34.7%, 2.8% and 4.9%, respectively (Fig 4A). Little is known about function of bacterial delta 6-desaturase. It has been reported that a cyanobacterial delta 6-desaturase is responsible for the conversion of linoleic acid (18:2) to gamma-linolenic acid (18:3 gamma) [47]. The *des6* gene product might be involved in the synthesis of C18:3 ω6, 9, 12c (gamma linolenic acid) which is present differently in three strains (0.825%, wild-type; 0.52%, Δ*adk* mutant; 0.44%, Δ*des6* mutant, Fig 4B). Measurements of unsaturated and saturated fatty acids indicate that membrane fluidity decreased in both mutant cells (Fig 4C). Fluidity index values of WT, Δ*adk* and Δ*des6* are 0.168, 0.136, and 0.122, respectively. Taken together, these results indicate that OA might increase cellular gentamicin uptake by changing membrane permeability.

### Influence of *adk* and *des6* on energy production

**Fig 5:**
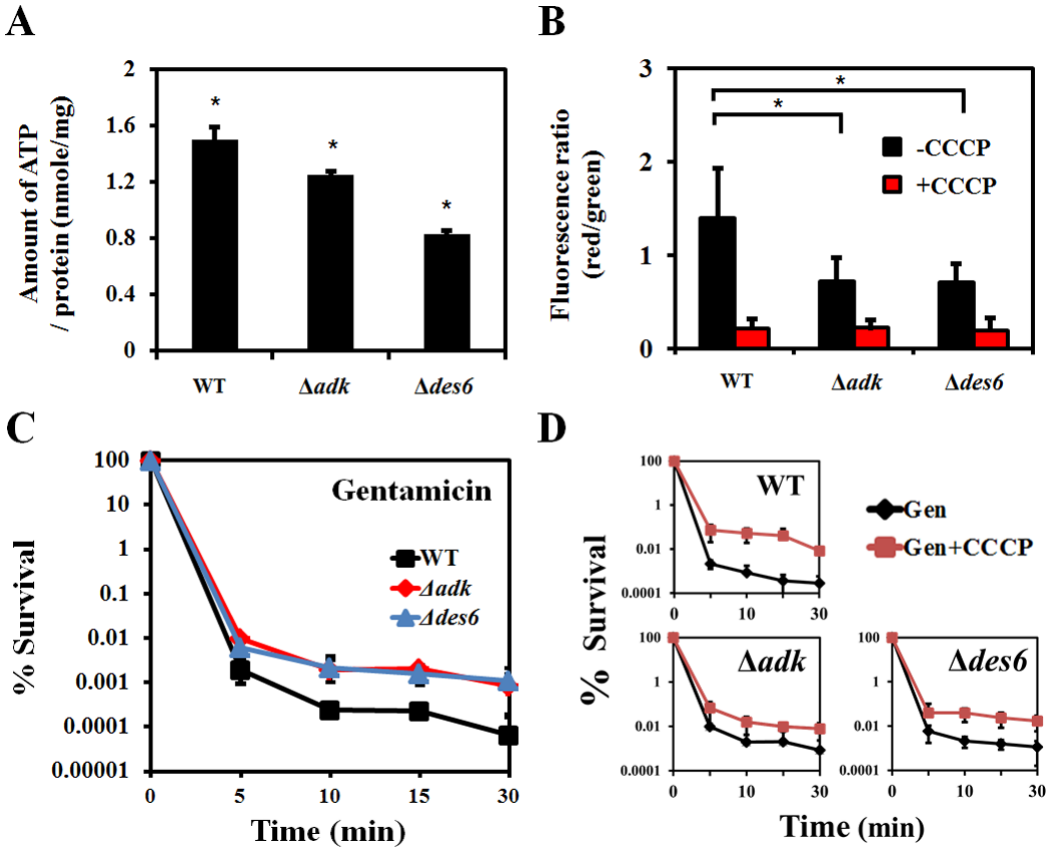
Energy-enabled aminoglycoside uptake and bacterial killing requires proton-motive force produced by *adk* and *des6* gene products. (A) The amount of ATP was determined by measuring luminescence, and each amount was calculated from the standard curve. Wild-type *Acinetobacter baumannii* ATCC17978 cells, Δ*adk* cells, and Δ*des6* cells were grown to the exponential phase and then treated with 64 μg/ml oleanolic acid for 30 min. (B) The fluorescence intensity of proton motive force is shown as the ratio of red to green. Flow cytometric analysis was conducted on wild-type cells, Δ*adk* cells, and Δ*des6* cells stained with 3 mM DiOC2(3) for 15 min with or without prior treatment with 500 μM carbonyl cyanide-m-chlorophenyl hydrazone (CCCP). (C) Effect of *adk* and *des6* genes on the resistance to gentamicin in wild-type cells, Δ*adk* cells, and Δ*des6* cells. We conducted survival tests for wild-type cells (black squares), Δ*adk* cells (red triangles), and Δ*des6* cells (blue squares) treated with the minimum inhibitory concentration of gentamicin (4 μg/ml) for 0, 5, 10, 15, or 30 min. (D) Effect of proton-motive force on bacterial killing. Cells treated with 10 μM CCCP were compared to cells treated with gentamicin alone; Gen, gentamicin. Data is represented as the average of two replicates and significance was measured by Student’s *t*-test (* P<0.05).

Many energy-related genes, including *adk* and ATP synthase F1 sector genes, were upregulated in our microarray (Table 2). Energy utilization has been reported to be important for the uptake of aminoglycosides. We hypothesized that the amount of ATP might be decreased in the both mutants and thus reduced the synergistic effect of OA on gentamicin. To test this hypothesis, we measured the amount of ATP in wild-type and mutant cells and found that levels of ATP were reduced in both mutants (Fig 5A). Although *adk* is known to be involved in ATP synthesis, the link between the deletion of *des6* and ATP reduction remains to be identified.

CCCP inhibits the proton gradient and thus prevents energy production; when cells are treated with CCCP, membrane potentials and ATP levels decrease [48]. When we measured membrane potentials using DiOC_2_(3), our data confirmed the effect of CCCP on membrane potentials and showed that, in the absence of CCCP, the membrane potentials of both mutants were lower than that of the wild-type cells (Fig 5B). It is worth noting that the amount of persister formation in both mutants with gentamicin treatment was greater than that in the wild-type strain, probably because of the reduced membrane potentials in the mutants (Fig 5C). The same phenomenon was observed with CCCP (Fig 5D); this observation indicates that lowering membrane potentials plays an important role for persister formation as well as gentamicin resistance.

## Discussion

Finding compounds that potentiate the effectiveness of antimicrobial agents on drug-resistant bacteria is an emerging issue in the battle against pathogenic bacteria. *A*. *baumannii* is resistant to almost all conventional antibiotics by a wide range of mechanisms [3] and can survive for prolonged periods on the surfaces of instruments in hospital settings [5]. Intrinsic antibiotic resistance together with its ability to form biofilms has made current medical approaches ineffective [28]. OA has potential antioxidative effects against fluoride-induced oxidative damage in mouse [49] and appears to influence the expression of stress response genes and the cysteine regulon in *E*. *coli* [50]. Although OA causes oxidative stress and several oxidative stress-related and DNA repair genes were upregulated in response to OA (Fig 1 and Table 2), the major redox-sensing regulon and related antioxidant genes were not highly induced under our experimental conditions. The reason for this discrepancy remains unclear; however, fine-tuning of gene regulation might induce the oxidative stress defense in *A*. *baumannii*, and this phenomenon could have not been identified under our single time-point microarray analysis.

Based on the known functions of the putative linoleoyl-CoA desaturase and adenylate kinase, the induction of these genes in response to OA might result in an increased permeability to gentamicin through changes in membrane lipid composition and in ATP synthesis (Figs 3–5). A similar conclusion was drawn when gentamicin uptake in the presence of bile acid, which is structurally similar to OA, was examined in a *Lactobacillus plantarum* strain [51], as it was though that the bile acid could permeabilize the membrane to gentamicin. Our results from the ANS and GTTR fluorescence assays support this conclusion (Fig 3).

The GTTR conjugate has proven to be useful in studying the endocytosis of aminoglycosides and their subsequent intracellular trafficking. Although the relative molecular mass of GTTR is larger than that of untagged gentamicin, its size does not affect its ability to permeate directly into the cytoplasm, and its distribution has been verified by using gentamicin immunocytochemistry. Other studies have shown that aminoglycoside uptake in human cells can be regulated by membrane potential, pH, extracellular cations (e.g., Ca^2+^, Gd^3+^, and La^3+^), and the nonspecific cation channel blocker ruthenium red, which is known to interact with a large number of proteins, such as ion channels [52].

OA is reported to inhibit biofilm formation in *Streptococcus* and *Actinomyces* strains [53]. However, in our study, when OA concentrations were increased, we observed an increase in biofilm formation (S4A Fig). These conflicting results could be due to strain differences, low concentration of OA and different solvent of OA between our study and other reports. Biofilm result is not necessarily to be positively related with MIC test. When MIC was determined, there is no biofilm in the microtiter plate. Our data demonstrated that OA could increase biofilm formation, which might be a characteristic of our *Acinetobacter* strain under our tested condition. To test whether *A*. *baumannii* was capable of motility, a 1 ml drop of an overnight culture was placed on LB media with agar concentrations ranging from 0.2 to 0.5%. Similar motility patterns were previously reported [54]. We found that ATCC17978 cells migrated across the plate from the central point of inoculation in a manner that resembled branching tentacles (S4B Fig). Treatment with either 32 μg/ml or 64 μg/ml OA drastically abolished motility, which was not seen in the presence of ethanol alone (S4B Fig). Our data show that OA increases biofilm formation and decreases motility (S4 Fig). The link between these observations and gentamicin uptake remains to be investigated. Taken together, our data demonstrate that the synergistic effect of OA with gentamicin in *A*. *baumannii* ATCC17978 could be due to increased uptake of aminoglycosides via increased energy production and membrane permeability. This study provides evidence of a new therapeutic potential for using OA-like compounds as antibiotic adjuvants in the treatment of MDR bacteria.

## Supporting Information

S1 Fig Time-kill curves under gentamicin plus OA. (DOCX)

S2 Fig Growth curves under OA treatment at different concentration. (DOCX)

S3 Fig Superoxide anion production using the NBT assay. (DOCX)

S4 Fig Biofilm formation and Motility of *A*. *baumannii* under OA condition. (DOCX)
