# Supplementary material for: Synergistic Effect of Oleanolic Acid on Aminoglycoside Antibiotics against Acinetobacter baumannii
Source: PLoS One. 2015 Sep 11;10(9):e0137751. doi: 10.1371/journal.pone.0137751 (PMC4567131; doi:10.1371/journal.pone.0137751)
Supplement: S1 Fig — (DOCX) [file pone.0137751.s001.docx]

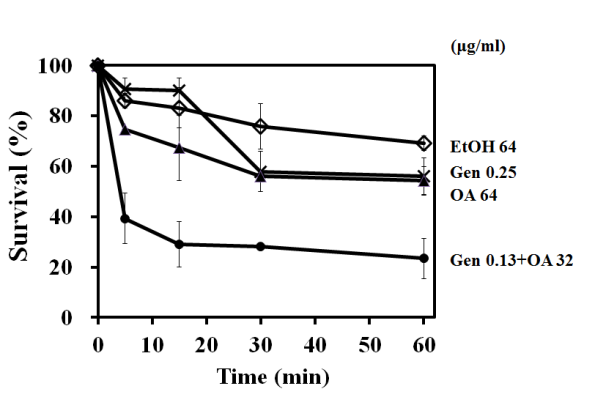


**S1 Fig. Time-kill curves under gentamicin plus OA*.*** Overnight culture of ATCC17978 (10^7^ CFUs/ml) were inoculated in 30 ml PBS supplemented with 1/8 MIC of gentamicin (0.25 μg/ml), 64 μg/ml OA and combination of gentamicin 1/16 MIC (0.13 μg/ml) with 32 μg/ml OA. The cultures were then incubated at room temperature with constant agitation (220 rpm). At each time point cells were harvested and washed twice in PBS. Each experimental point represents the mean of 2 replicates.
