# Supplementary material for: Synergistic Effect of Oleanolic Acid on Aminoglycoside Antibiotics against Acinetobacter baumannii
Source: PLoS One. 2015 Sep 11;10(9):e0137751. doi: 10.1371/journal.pone.0137751 (PMC4567131; doi:10.1371/journal.pone.0137751)
Supplement: S2 Fig — (DOCX) [file pone.0137751.s002.docx]

**
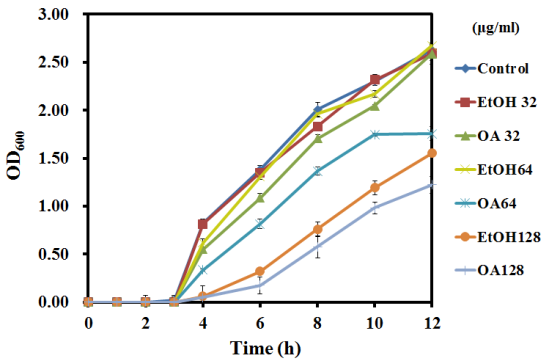
**

**S2 Fig. Growth curves under OA treatment at different concentration.** Overnight cultures of ATCC17978 were diluted to 10^6^ CFUs/ml and incubated for 12 h at 37°C. Growth curves in the presence of different OA concentrations (32, 64, 128 μg/ml) and ethanol solvent were measured. Growth was monitored by measuring the OD_600_ of the cultures using a BioPhotometer (Eppendorf). Each experimental point represents the mean of 3 replicates.
