# Supplementary material for: Synergistic Effect of Oleanolic Acid on Aminoglycoside Antibiotics against Acinetobacter baumannii
Source: PLoS One. 2015 Sep 11;10(9):e0137751. doi: 10.1371/journal.pone.0137751 (PMC4567131; doi:10.1371/journal.pone.0137751)
Supplement: S3 Fig — (DOCX) [file pone.0137751.s003.docx]

**
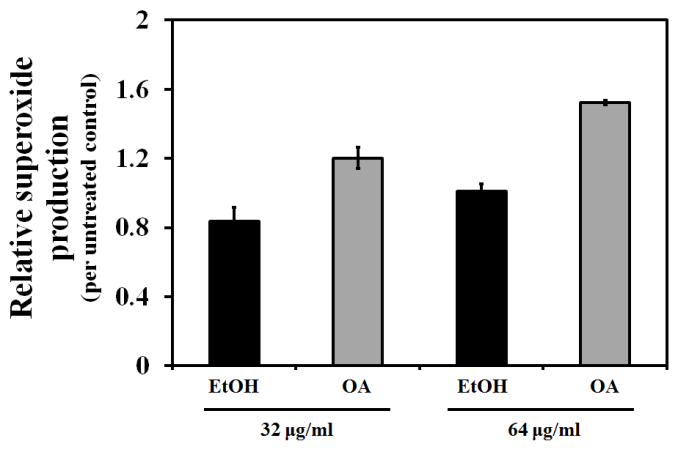
**

**S3 Fig.** **Superoxide anion production using the NBT assay**. Exponentially grown cells (1 ml) were incubated with either OA or ethanol for 30min and then 0.5 mL of NBT was added for 30 min at 37°C. 0.1 mL of 0.1 M HCl was added, and the tubes were centrifuged. The separated pellets were treated with 0.4 mL of dimethyl sulfoxide to extract the reduced NBT; finally, 0.8 mL of PBS (pH 7.5) was added. OD_575_ of superoxide production was measured by spectrophotometer. Each experimental point represents the mean of 3 replicates.
