# Supplementary material for: Synergistic Effect of Oleanolic Acid on Aminoglycoside Antibiotics against Acinetobacter baumannii
Source: PLoS One. 2015 Sep 11;10(9):e0137751. doi: 10.1371/journal.pone.0137751 (PMC4567131; doi:10.1371/journal.pone.0137751)
Supplement: S4 Fig — (DOCX) [file pone.0137751.s004.docx]

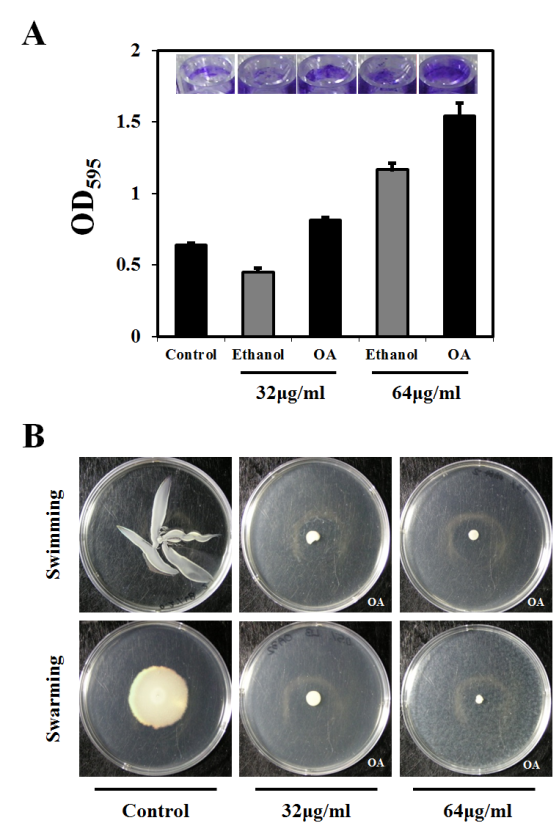


**S4 Fig. Biofilm formation and Motility of *A. baumannii* under OA condition.** (A) Biofilm assay using 1/5 diluted LB, at 37°C for 24 h. Biofilm formation was analyzed as described previously [30]. (B) An overnight culture of *A. baumannii* was spotted on 0.2%, 0.5% LB agar plate containing 32 μg/ml and 64 μg/ml of OA, respectively. After 1 μl drop was placed on the surface agar plate, the plates were incubated at 37°C for 12 h. Each experimental point represents the mean of 3 replicates.
